# GAN-Based Cross-Modality Brain MRI Synthesis: Paired Versus Unpaired Training and Comparison with Diffusion and Transformer Models

**DOI:** 10.3390/biomimetics11030175

**Published:** 2026-03-02

**Authors:** Behnam Kiani Kalejahi, Sebelan Danishvar, Mohammad Javad Rajabi

**Affiliations:** 1 Department of Computer Science, School of Engineering, Central Asian University, Tashkent 111211, Uzbekistan; 2 Faculty of Data Science and Information Technology, INTI International University, Persiaran Perdana BBN Putra Nilai, Nilai 71800, Malaysia; 3 Department of Electronic and Computer Engineering, Brunel University, Uxbridge UB83PH, UK

**Keywords:** generative adversarial networks, CycleGAN, diffusion models, human health, brain tumour

## Abstract

Incomplete or faulty MRI sequences are common in clinical practice and can impair AI-based analyses that rely on complete multi-contrast data. The relative effectiveness of classical generative adversarial networks (GANs) versus modern diffusion and transformer-based models for clinically usable MRI synthesis remains unclear. This study evaluates cross-modality MRI synthesis using the BraTS 2019 brain tumour dataset, focusing on T1-to-T2 translation. We assess paired and unpaired CycleGAN models and compare them with two stronger but computationally intensive baselines, a conditional denoising diffusion probabilistic model (DDPM) and a transformer-enhanced GAN, using identical data splits and preprocessing pipelines. Inter-modality correlation was evaluated to estimate the achievable similarity between modalities. Conceptually, modality synthesis may be viewed as a representation-learning approach that compensates for missing imaging information by reconstructing clinically relevant features from available contrasts. Paired CycleGAN achieved correlations of r≈0.92–0.93  and SSIM ≈0.90–0.92, approaching natural T1–T2 correlation (r≈0.95) while maintaining very fast inference (<50 ms/slice). Unpaired CycleGAN achieved r≈0.74–0.78 and SSIM ≈0.82–0.85, producing clinically interpretable reconstructions without voxel-level supervision. DDPM achieved the highest fidelity (SSIM ≈0.93–0.95, r≈0.94) but required substantially greater computational resources, while transformer-enhanced GAN performance was intermediate. Qualitative analysis showed that CycleGAN and DDPM best preserved tumour and tissue boundaries, whereas unpaired CycleGAN occasionally over-smoothed subtle lesions. These findings highlight the trade-off between fidelity and efficiency in cross-modality MRI synthesis, suggesting paired CycleGAN for time-sensitive clinical workflows and diffusion models as a computationally expensive accuracy upper bound.

## 1. Introduction

Artificial intelligence (AI) and deep generative models have transformed medical image analysis, enabling advanced capabilities in synthesis, harmonisation, augmentation, and automated diagnostic support. Generative adversarial networks (GANs), first introduced by Goodfellow et al. [1], have become foundational to these developments. GANs rely on an adversarial interplay between a generator and a discriminator to synthesise highly realistic images, a framework that has since influenced numerous applications in computer vision, natural language processing, and, increasingly, medical imaging [2,3,4].

Magnetic resonance imaging (MRI) plays a pivotal role in neuroimaging because of its excellent soft tissue contrast and versatility. T1-weighted images provide high anatomical detail, while T2-weighted images highlight fluid alterations and inflammatory processes. The complementary nature of these modalities makes multimodal acquisition essential for tumour detection, oedema assessment, and surgical planning. However, in real-world clinical settings, multimodal MRI is frequently incomplete due to motion artefacts, acquisition errors, time limitations, or variations in imaging protocols [1,5]. Missing contrasts limit radiological interpretation and impair the performance of AI pipelines that rely on complete and well-balanced multi-contrast datasets [6,7].

From a computational perspective, cross-modality MRI synthesis can be interpreted as a learned mapping between imaging domains rather than solely as a conceptual analogy to biological perception. Let X denote the source modality (e.g., T1-weighted MRI) and Y the target modality (e.g., T2-weighted MRI), where a generator $G_{\theta }:X\to Y$ learns a mapping preserving anatomical structure while translating contrast information. Within this framework, the notion of perceptual augmentation corresponds to representation learning that compensates for missing imaging channels by inferring clinically meaningful features from available inputs. This interpretation aligns the bionic analogy with a mathematically grounded mapping process, where generative models serve as computational mechanisms for cross-contrast sensory substitution constrained by anatomical consistency. Related abstraction-driven perspectives have also been explored in neuromorphic computing, where synapse-inspired systems and memristive devices are used to model adaptive signal transformation and information routing, offering conceptual parallels to learned representation mapping in generative models. This interpretation motivates evaluating synthesis models not only by visual realism but also by their ability to preserve clinically relevant representations across modalities.

Synapse-inspired memristive systems, originally theorised by Chua [8] and physically realised by Strukov et al. [9], have been widely studied as hardware substrates for adaptive signal transformation in neuromorphic circuits [5,10].

Cross-modality MRI synthesis aims to estimate a missing sequence (e.g., generating T2 from T1) using learned transformations between modalities. Early deep learning approaches relied on supervised frameworks such as conditional GANs (cGANs) [11,12], regression-based synthesis [13], or sparse-coding methods [14]. Nie et al. [6] explored GAN-based MRI-to-CT translation, while Dar et al. [15] demonstrated conditional synthesis of T2 from T1 using paired MRI data. Welander et al. [16] later compared CycleGAN and UNIT for unpaired multi-contrast MRI translation, showing that cycle-consistency enables robust synthesis even without aligned T1–T2 pairs.

Cycle-consistent GANs (CycleGAN), introduced by Zhu et al. [17], remain one of the most influential architectures for unpaired medical image translation. Their ability to operate without precisely registered pairs is particularly advantageous for clinical MRI, where paired and spatially aligned datasets are scarce or inconsistent across centres [18,19]. CycleGAN has since been widely adopted for MRI translation tasks involving the brain, abdomen, and spine [20], and has also been studied for artefact reduction, intensity harmonisation, and data augmentation.

Recent advances (2022–2025) have expanded beyond GANs to include diffusion models, transformer-based approaches, and 3D volumetric generative frameworks. Cross-conditioned diffusion models [21] and target-guided diffusion models [22] have demonstrated state-of-the-art performance for multi-contrast medical image translation. Cascaded multi-path diffusion frameworks [23] and adaptive latent diffusion systems [24] have further improved structural fidelity and robustness to distribution shifts. In parallel, transformer-enhanced GANs have shown improved global context modelling in MRI synthesis and reconstruction [25,26]. Volumetric GANs such as IGUANe and multi-resolution 3D GANs [27] have pushed the boundaries of 3D synthesis, offering improved spatial coherence across slices.

Despite these promising developments, many recent models depend on large-scale training datasets, high-end GPUs, and time-consuming inference proceduresparticularly diffusion-based approaches, which may require hundreds of denoising steps per generated image. This substantially limits their practical adoption in resource-constrained clinical environments. Lightweight architectures such as CycleGAN therefore remain attractive due to their computational efficiency, lower hardware requirements, and ability to operate with limited paired data, making them well suited for real-world deployment in resource-constrained clinical workflows.

### 1.1. Gap and Motivation

Although numerous studies have examined CycleGAN for MRI translation, several important questions remain insufficiently explored:

1. How does CycleGAN performance differ between paired and unpaired training when evaluated on the same dataset under identical preprocessing and evaluation protocols?
2. How does CycleGAN compare quantitatively with modern diffusion-based and transformer-enhanced generative models under identical experimental conditions?
3. To what extent do these models preserve clinically relevant anatomical and pathological structures, including tumour morphology and tissue boundaries?
4. What are the trade-offs between computational cost, inference latency, and image fidelity that determine real-world clinical deployability?

Despite increasing interest in AI-based modality synthesis, few studies explicitly frame cross-modality translation as a bionic perceptual augmentation problem, where artificial systems compensate for missing biological sensing within practical clinical workflows. In particular, the relationship between model fidelity, computational efficiency, and functional usability as an artificial perceptual substitute remains underexplored.

These gaps motivate the present study, which evaluates CycleGAN-based cross-modality MRI synthesis from both a quantitative benchmarking perspective and a bionics-inspired deployment-oriented viewpoint.

### 1.2. Contributions

This paper presents a comprehensive evaluation of CycleGAN for T1↔T2 brain MRI synthesis in both paired and unpaired settings using the BraTS 2019 dataset. The key contributions are as follows:

1. **Paired vs. unpaired supervision under identical conditions.** We provide, to our knowledge, one of the first systematic comparisons of paired and unpaired CycleGAN training for T1↔T2 brain MRI synthesis using the same dataset, same preprocessing, and same evaluation protocol, quantifying how supervision mode affects structural fidelity and lesion preservation.
2. **Upper-bound-aware evaluation of cross-modality synthesis.** We explicitly measure the intrinsic correlation between real T1 and real T2 images in BraTS 2019 and use it as an empirical upper bound for achievable cross-modality similarity. All model correlations are interpreted relative to this bound, offering a statistically grounded view of “how close to optimal” each method is.
3. **Benchmarking CycleGAN against modern diffusion and transformer-based models.** CycleGAN (paired/unpaired) is benchmarked against a conditional DDPM and a transformer-enhanced GAN under identical training and test conditions, enabling a fair comparison of architecture families rather than single, isolated models.
4. **Deployment-focused analysis of computational efficiency.** Beyond image quality metrics, we systematically compare training time, inference latency, and hardware requirements, highlighting trade-offs between fidelity and deployability that are critical for real-world clinical workflows.
5. **Clinical interpretability with lesion- and tissue-focused analysis.** We complement global similarity metrics (SSIM, PSNR, MAE, correlation) with qualitative, radiologically motivated assessment of tumour morphology, oedema patterns, and grey–white matter contrast to assess the clinical plausibility of synthetic contrasts.
6. Bionics-oriented interpretation of MRI synthesis. We frame cross-modality MRI translation as a bionic perceptual compensation mechanism, where generative models function as artificial sensory surrogates for missing or corrupted biological imaging channels.

## 2. Related Works

### 2.1. Early Approaches to Cross-Modality Medical Image Synthesis

Early research on medical image synthesis used classical regression and sparse-coding techniques to estimate one imaging modality from another. Jog et al. [13] applied random forest regression to synthesise MR images, while Huang et al. [14] proposed joint convolutional sparse coding for simultaneous super-resolution and cross-modality reconstruction. Although these approaches demonstrated the feasibility of contrast-to-contrast estimation, they required carefully aligned paired datasets and struggled to preserve fine anatomical structures.

Subsequent work introduced conventional CNN-based synthesis methods, including weakly supervised 3D reconstruction [14], early 3D CNN-based diagnostic models for lesion detection [28], and cross-modality methods supporting downstream tasks such as registration and segmentation [18]. These early systems were limited by their inability to synthesise high-fidelity images with realistic texture, motivating the adoption of deep generative models.

### 2.2. GAN-Based Image-to-Image Translation in Medical Imaging

The introduction of generative adversarial networks (GANs) by Goodfellow et al. [1] triggered rapid development of deep generative techniques for medical imaging. Conditional GANs (cGANs) such as Pix2Pix [11,29], based on the framework of Mirza and Osindero [12], enabled paired image-to-image translation and were soon applied to multi-contrast MRI and MRI-CT synthesis. Subsequent refinements and stability improvements to adversarial training in medical imaging contexts have also been explored [30]. Nie et al. [31] demonstrated MRI-to-CT translation using context-aware GANs, and Dar et al. [15] generated T2-weighted MR images from T1-weighted scans using a paired cGAN framework. Yang et al. [18] similarly employed deep image-to-image translation for cross-modality MRI registration and segmentation.

Unpaired image translation emerged with DualGAN [32] and related dual-learning frameworks, enabling learning of bidirectional mappings without aligned training pairs. Welander et al. [16] compared CycleGAN and UNIT, finding that unpaired translation models can produce clinically plausible multi-contrast MR images.

GAN-based models have since been widely adopted for medical synthesis, augmentation and domain adaptation tasks, including data augmentation approaches such as DAGAN [9]. Applications range from skin lesion generation [3,29] and brain tumour augmentation [4,33] to pixel-level domain transfer [34] and unsupervised cross-modal representation learning [35,36,37].

Although Pix2Pix and U-Net-based conditional GANs are widely used as standard baselines for paired image translation, paired CycleGAN was retained in this study primarily to maintain architectural consistency between paired and unpaired settings. The cycle consistency objective introduces an additional structural constraint by enforcing approximate invertibility between modalities, which acts as a regulariser and may improve mapping stability in data-limited scenarios. This design choice allows for controlled comparison of supervision regimes within a unified framework and should not be interpreted as a claim of superiority over Pix2Pix-style approaches.

### 2.3. CycleGAN for Unpaired Modality Translation

CycleGAN, introduced by Zhu et al. [17], remains one of the most influential models for unpaired medical image synthesis. CycleGAN enforces cycle consistency, enabling forward and backward transformations (e.g., T1→T2→T1) and preserving anatomical structure even without paired supervision. This makes CycleGAN particularly suitable for MRI, where acquiring perfectly aligned T1–T2 pairs is often infeasible.

Several works have applied CycleGAN-style architectures to MRI translation [38]. Welander et al. [16] demonstrated unpaired T1–T2 synthesis feasibility, while Vemulapalli et al. [19] performed unsupervised cross-modal synthesis of subject-specific MR scans. Kim et al. [20] used GANs for CT-to-T2 translation of the lumbar spine, showing strong clinical potential. GAN variants such as Wasserstein GAN [20], StackGAN [19,21], and perceptual similarity GANs [26] have also contributed architectural insights for high-fidelity synthesis.

Despite these advances, most prior studies focus exclusively on either paired or unpaired training, rarely comparing both modes under identical conditions on the same dataset, which is a key motivation for the present study.

### 2.4. Extensions: 3D GANs, Transformer-Based Models and Diffusion Models

As computational capabilities increased, researchers explored volumetric and multi-resolution 3D GANs for improved spatial coherence across slices. Zhou et al. [27] introduced multi-resolution guided 3D GANs, and Kim et al. [24] proposed adaptive latent diffusion for 3D medical translations. These volumetric architectures overcome slice inconsistencies but require significantly more memory and computational resources.

Transformer-based architectures have recently become central to medical image analysis, primarily due to their capacity to model long-range global context. Swin-UNETR and related transformer–UNet hybrids [25] demonstrated strong performance in 3D MRI segmentation, while self-supervised transformer frameworks [26] improved multimodal feature representation learning. Transformer ideas have also been incorporated into GAN generators, improving anatomical consistency in generative tasks.

Diffusion models represent the latest generation of high-fidelity generative models. Denoising diffusion probabilistic models (DDPMs) [39] have been adapted for MRI synthesis, as shown by Pinaya et al. [40], while cross-conditioned diffusion [21], target-guided diffusion [22], and cascaded multi-path diffusion systems [23] have further advanced cross-modality translation. Comprehensive surveys [41] highlight diffusion models’ excellent realism and robustness, though they typically require significantly longer inference times and larger computational budgets than GAN-based alternatives.

### 2.5. Summary and Motivation for This Study

The literature demonstrates a progression from classical sparse-coding methods [13,14] to paired cGANs [11,12,15,29,31], unpaired CycleGAN/UNIT models [16,17,19,32], and finally transformer-enhanced and diffusion-based approaches [21,22,23,24,25,26,27,39]. Table 1 summarises the main generative model families used in cross-modality medical image synthesis, highlighting their strengths and limitations.

However, several gaps remain:

- Paired vs. unpaired CycleGAN performance has rarely been compared directly using the same dataset and consistent evaluation metrics.
- Quantitative benchmarking of CycleGAN against diffusion and transformer-enhanced generative models under identical experimental conditions is lacking.
- The trade-off between computational efficiency (GANs) and image fidelity performance (diffusion/transformer models) remains underexplored.
- Few studies evaluate whether models preserve clinically meaningful lesion/tissue features, beyond generic similarity metrics.

This study addresses these gaps by

1. Evaluating CycleGAN in both paired and unpaired regimes for T1↔T2 MRI synthesis.
2. Benchmarking CycleGAN against DDPM-based diffusion and transformer-enhanced GAN baselines.
3. Comparing performance using SSIM, PSNR, MAE, and correlation metrics, alongside visual and clinical interpretability analyses.

### 2.6. Human Feedback for Model Alignment

Recent research in large-scale model training has demonstrated the value of human feedback signals for behaviour alignment and internal representation refinement. For example, Ouyang et al. introduced a framework in which reinforcement learning from human feedback (RLHF) is used to optimise a language model to follow natural language instructions, resulting in performance improvements on subjective and multi-step reasoning tasks relative to standard supervised fine-tuning [43]. Similarly, Wong et al. explored the alignment of crowd-sourced human feedback for reinforcement learning in code generation tasks, showing that human preferences can guide model policy updates that correlate with quantitatively better output quality [44].

While these works focus on language domains, the underlying principle of feedback-informed optimisation—combining explicit human or proxy evaluations with model parameter updates, offers a conceptual basis for transformer-style architectures in other modalities, including medical image translation. In our context, such signals could inform attention map refinement or feature representation adaptation during training, complementing purely adversarial and reconstruction losses.

### 2.7. Implication for Transformer Modules

Specifically, transformer attention weights could be indirectly optimised not only via gradient descent on prediction losses, but also through feedback-derived reward signals or preference-conditioned loss terms that prioritise perceptual fidelity, anatomical consistency, or task-specific utility as judged by experts or downstream tasks.

## 3. Methodology

### 3.1. Dataset and Preprocessing

This project uses the BraTS 2019 brain tumour dataset, which provides multimodal MRI volumes (including T1- and T2-weighted scans) acquired from several centres and harmonised by the organisers. This dataset has become a widely adopted benchmark for tasks such as brain tumour segmentation and cross-modality synthesis because of its high-quality annotations and standardised preprocessing pipeline [7].

For this study, we utilised 1113 volumetric MRI scans from the BraTS 2019 dataset, including both T1-weighted and T2-weighted images. All volumes were resampled to 1 mm isotropic resolution and skull-stripped using the official BraTS preprocessing pipeline. Each 3D volume was then decomposed into 2D axial slices only, with each slice resized to 256 × 256 grayscale images. Slices containing only background were discarded to avoid trivial samples. To account for scanner- and site-dependent intensity variability, z-score normalisation was applied independently to each volume by subtracting the mean and dividing by the standard deviation of all brain voxels.

The data were split at the subject level into training (70%), validation (15%), and test (15%) sets, ensuring that no patient contributed slices to more than one subset and thereby preventing information leakage between training and evaluation. Prior to augmentation, this resulted in approximately 713 volumetric scans for training, 200 for validation, and 200 for testing, balanced across imaging modalities. Following axial slicing and data augmentation, the effective dataset size was expanded to approximately 24,000 slices for training and 11,000 slices for testing across all experiments.

After splitting patients into training, validation, and test sets, we constructed paired and unpaired datasets as follows: For the paired setting, each T1 slice was matched with the spatially aligned T2 slice from the same subject and axial position. For the unpaired setting, T1 and T2 slices were sampled independently from different subjects within the training set, following Zhu et al. [17], such that no voxel-wise anatomical correspondence was available during training. Importantly, the validation and test sets remained strictly paired and subject-exclusive for all experiments.

The use of the BraTS dataset was motivated by the complementary nature of T1- and T2-weighted MRI contrasts. T1-weighted images highlight anatomical structure, while T2-weighted images emphasise fluids and inflammatory processes. This complementarity makes T1–T2 synthesis clinically relevant, particularly in scenarios where one modality is missing or corrupted.

Although MRI data are inherently volumetric, 2D slice-based models were used in this study to enable computationally feasible training and fair comparison across diverse model families under identical hardware and preprocessing conditions. Using 2D inputs reduces memory requirements and allows for consistent benchmarking of GAN-, transformer-, and diffusion-based approaches without introducing confounding differences in model scale or training complexity.

### 3.2. Data Augmentation

To enhance robustness and mitigate overfitting, augmentation was applied only to the training set. Each slice was transformed with one or more of the following, applied with 0.5 probability:

- Random horizontal/vertical flips;
- Small rotations (±10°);
- Random cropping and resizing (90–100% of FOV);
- Intensity scaling and minor brightness shifts;
- Gaussian noise injection (σ = 0.01).

In paired training, augmentations were applied identically to T1 and T2 slices to maintain alignment. No augmentation was applied to validation or test sets.

### 3.3. CycleGAN Architecture

CycleGAN enables unpaired image-to-image translation between two modalities:

- Domain X = T1-weighted MRI
- Domain Y = T2-weighted MRI

It learns two generators:

- G: X → Y (T1→T2)
- F: Y → X (T2→T1)

And two discriminators:

- D_X for distinguishing real vs. synthetic T1
- D_Y for distinguishing real vs. synthetic T2

CycleGAN does NOT use random noise as input.

Unlike standard GANs [3], CycleGAN takes a real image as input, and the mapping is deterministic.

#### 3.3.1. Generator Architecture

Each generator uses the standard ResNet-based CycleGAN backbone [10], consisting of

- Two downsampling convolutional blocks;
- Nine residual blocks (ResNet-9);
- Two upsampling blocks.

#### 3.3.2. Discriminator Architecture

PatchGAN discriminators classify 70 × 70 patches as real or fake [11], improving stability and texture consistency.

#### 3.3.3. Loss Functions

CycleGAN optimises a combination of

1. Adversarial loss (LSGAN);
2. Cycle-consistency loss:

(1) $$L_{cyc}=∥F(G(x))-x{∥}_{1}+∥G(F(y))-y{∥}_{1}$$

where

- x∈X is an input image from domain X (e.g., T1 MRI);
- y∈Y is an input image from domain Y (e.g., T2 MRI);
- G:X→Y is the forward generator;
- F:Y→X is the backward generator;
- $∥\cdot {∥}_{1}$ denotes the L1 norm (Mean Absolute Error over pixels).

3. Identity loss (optional, improves contrast preservation)

The final loss is

(2) $$L=L_{GAN}(G,D_{Y})+L_{GAN}(F,D_{X})+\lambda_{cyc}L_{cyc}+\lambda_{id}L_{id}$$

with λ_{cyc} = 10 and λ_{id} = 5, as commonly used in the MRI translation literature [4,8].

- L: total objective function;
- $L_{GAN}(G,D_{Y})$: adversarial loss for generator G:X→Y and discriminator $D_{Y}$;
- $L_{GAN}(F,D_{X})$: adversarial loss for generator F:Y→X and discriminator $D_{X}$;
- $L_{cyc}$: cycle-consistency loss;
- $L_{id}$: identity preservation loss;
- $\lambda_{cyc}$: cycle-consistency weighting coefficient (typically 10);
- $\lambda_{id}$: identity loss weighting coefficient (typically 5).

All convolutional layers in the generators and discriminators used instance normalisation rather than batch normalisation, consistent with prior work on image-to-image translation and medical MRI synthesis [15,16,17]. Instance normalisation was found to stabilise training under small batch sizes and heterogeneous intensity distributions.

Discriminators were implemented as PatchGANs without spectral normalisation. In our experiments, we did not observe training instabilities that would necessitate spectral norm, and omitting it kept the architecture closer to the widely adopted baseline CycleGAN configuration.

**Figure 1:**
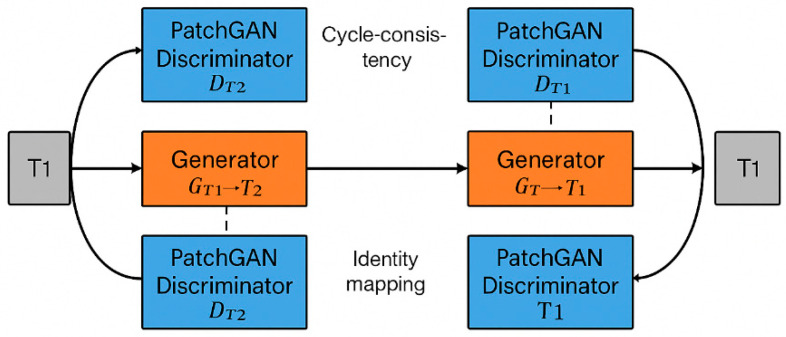
CycleGAN architecture for cross-modality MRI synthesis.

An overview of the CycleGAN architecture used for cross-modality MRI synthesis is illustrated in Figure 1.

The framework consists of two generators learning bidirectional mappings between T1- and T2-weighted MRI, supervised by PatchGAN discriminators and constrained by cycle consistency to preserve anatomical structure. This architecture supports both paired and unpaired training without requiring voxel-wise correspondence.

### 3.4. Training Settings (Paired vs. Unpaired)

- Paired training:

Each T1 slice is matched with the corresponding T2 slice from the same subject. Loss functions remain unchanged, but the mapping benefits from spatial alignment. The detailed training procedure for paired CycleGAN is summarised in Algorithm 1.

- *Unpaired training:*

T1 and T2 slices come from different subjects.

Cycle consistency enforces structural preservation without paired supervision.

All models were trained for 200 epochs using Adam (β_1_ = 0.5, β_2_ = 0.999) with a learning rate of 2 × 10^−4^ for the first 100 epochs and linear decay afterwards.

Training was performed on a single NVIDIA GPU (RTX-series), demonstrating clinical feasibility. The full optimisation procedure for the unpaired setting is outlined in Algorithm 2.

### 3.5. Diffusion Model (DDPM) Baseline

We implemented a conditional denoising diffusion probabilistic model following Ho et al. [39] and recent MRI translation works [21,22,23]. The iterative denoising procedure used during DDPM inference is summarised in Algorithm 3.

A DDPM models image generation as a gradual denoising process:

1. Forward process:

Noise is added to T2 images over T steps:

(3) $$q(x_{t}|x_{t-1})=N(\sqrt{\alpha_{t}}x_{t-1},(1-\alpha_{t})I)$$

- $x_{t-1}$: image (or latent) at diffusion step t−1;
- $x_{t}$: image (or latent) at diffusion step t after adding noise;
- N(⋅;μ,Σ): Gaussian distribution with mean μ and covariance Σ;
- $\alpha_{t}\in (0,1)$: noise schedule coefficient controlling how much signal is kept at step t;
- I: identity matrix (so covariance is isotropic);
- $\left(1-\alpha_{t}\right)$: variance of the injected Gaussian noise at step t.

2. Reverse process:

A U-Net predicts the noise at each step conditioned on the source modality (T1).

Thus, the model synthesises T2 as

(4) $$\hat{y}=\mathrm{DDPM}(x\_T1)$$

- $x_{T1}$: input T1-weighted MRI slice;
- $\hat{y}$: synthesised T2-weighted MRI slice;
- DDPM(⋅): conditional diffusion model mapping from source modality (T1) to target modality (T2).

Inference requires 200–400 denoising steps, making DDPM computationally heavier but often more precise.

### 3.6. Transformer-Enhanced GAN Baseline

To compare CycleGAN with a modern architecture, we implemented a transformer-augmented generator inspired by Swin-UNETR transformers [25], transformer–GAN hybrids [37], and recent multimodal MRI synthesis methods.

The model replaces the CycleGAN generator’s bottleneck with

- A Swin Transformer encoder for long-range context;
- Skip connections to convolutional decoding layers;
- Adversarial training similar to Pix2Pix [11].

This baseline enables evaluation of how global attention impacts MRI synthesis.

### 3.7. Evaluation Metrics

We explicitly define all metrics:

- Structural Similarity Index (SSIM): Measures structural fidelity, contrast, and luminance similarity.
- Peak Signal-to-Noise Ratio (PSNR): Evaluates pixel-level reconstruction quality.
- Mean Absolute Error (MAE): Captures average per-pixel deviation.

#### Pearson Correlation Coefficient (r)

Used to measure linear similarity between synthetic (Ŷ) and ground truth (Y) slices:

(5) $$r=\frac{\sum_{i}\left(Y_{i}-\bar{Y})({\hat{Y}}_{i}-\bar{\hat{Y}}\right)}{\sqrt{\sum_{i}(Y_{i}-\bar{Y}{)}^{2}}\sqrt{\sum_{i}({\hat{Y}}_{i}-\bar{\hat{Y}}{)}^{2}}}$$

- $Y_{i}$: ground truth voxel intensity at index i;
- ${\hat{Y}}_{i}$: synthesised voxel intensity at index i;
- $\bar{Y}$: mean intensity of ground truth image over evaluated voxels;
- $\bar{\hat{Y}}$: mean intensity of synthesised image over evaluated voxels;
- N: number of voxels included in the evaluation mask;
- r∈[−1,1]: Pearson correlation coefficient.

Values range from −1 to 1.

- Higher r indicates stronger anatomical similarity.

Most importantly:

r is always computed between synthetic images and their real target slices.

Additionally, we computed the natural correlation between real T1 and real T2, providing an upper bound for achievable cross-modality similarity.

This value serves as an empirical upper bound on achievable cross-modality similarity for the BraTS dataset, rather than a theoretical maximum, as it reflects intrinsic biological and acquisition-related differences between T1- and T2-weighted MRI.

Pearson correlation coefficient between the synthesised image and reference image was determined on anatomically relevant regions to reduce inflation due to background voxels. The inflation occurs due to large areas of uniform background. The binary evaluation mask was created by combining non-zero voxels from source and reference image post-preprocessing and intensity normalisation. The correlation was determined only on this evaluation mask. The correlation measure was determined on a per-volume basis and then averaged across all subjects.

In addition to Pearson correlation, Mutual Information (MI) is discussed as a complementary modality-invariant measure of statistical dependence that can capture non-linear cross-modality relationships and is less sensitive to contrast inversion than linear correlation. However, MI was not included in the quantitative benchmarking reported in this study and is suggested as a useful metric for future evaluations.

Model selection and early stopping were performed using the validation split, while all reported PSNR and SSIM values correspond to the independent test split. Model selection and early stopping were performed using the validation split, while all reported quantitative metrics (PSNR, SSIM, MAE, and correlation) correspond to evaluation on the held-out test split.

### 3.8. On the Generative Capacity of CycleGAN

CycleGAN performs conditional translation, not unconditional generation:

- It cannot create new anatomical variations beyond what is present in the input modality.
- It requires an existing T1 to produce a T2, or vice versa.

Thus, CycleGAN is suitable for modality completion, data harmonisation, and augmentation of multimodal datasets, but not for de novo dataset creation.

From a bionics standpoint, CycleGAN acts as a deterministic artificial sensory mapping rather than a stochastic image generator. Unlike unconditional generative models that sample from a latent distribution, CycleGAN performs a conditional and deterministic transformation from an input modality to a target modality, producing a consistent output for a given input image.

This determinism is critical for bionic reliability in clinical settings, where artificial perceptual systems are expected to behave predictably and reproducibly. In the context of modality completion, deterministic mappings ensure that identical anatomical inputs yield identical synthetic outputs, thereby supporting repeatable clinical interpretation and downstream decision-making. Conversely, stochastic variation in reconstructed images may undermine trust in artificial sensory augmentation systems by introducing non-biological variability that is unrelated to patient anatomy.

Furthermore, hallucination risk represents a key safety concern from a bionics perspective. As artificial perceptual substitutes, modality synthesis models must prioritise faithful reconstruction of biologically plausible structures over visual realism. Spurious textures, fabricated lesions, or suppressed pathological features can mislead clinical interpretation in a manner analogous to faulty sensory prostheses. Cycle consistency constraints in CycleGAN partially mitigate this risk by enforcing anatomical reversibility between modalities, thereby limiting the introduction of non-existent structures and supporting safer deployment in clinical workflows.

### 3.9. Downstream Evaluation: Tumour Segmentation with Synthetic T2

#### 3.9.1. Rationale

Global similarity metrics such as SSIM and PSNR do not fully capture the clinical utility of synthetic MRI modalities. To assess whether generated images preserve information relevant for diagnostic tasks, we conducted a downstream evaluation using brain tumour segmentation as a representative and clinically meaningful task.

Tumour segmentation is highly sensitive to T2-weighted contrast, particularly for delineating oedema and non-enhancing tumour regions. If a synthetic T2 image preserves pathology-relevant features, a segmentation model trained on real T2 images should generalise to synthetic T2 with minimal performance degradation.

#### 3.9.2. Experimental Setup

We trained a standard 2D U-Net architecture on real T2 images from the BraTS 2019 training set using the provided ground truth tumour masks. The network was optimised using Dice loss with Adam optimisation and early stopping on a held-out validation set.

At test time, segmentation performance was evaluated under four conditions:

1. Real T2 (reference upper bound);
2. Synthetic T2 generated by paired CycleGAN;
3. Synthetic T2 generated by unpaired CycleGAN;
4. Synthetic T2 generated by the DDPM diffusion model.

All evaluations were performed on the same held-out BraTS 2019 test subjects to ensure fair comparison. No further fine-tuning of the segmentation network was performed on synthetic images.

#### 3.9.3. Evaluation Metric

Segmentation performance was quantified using the Dice Similarity Coefficient (DSC) between predicted tumour masks and ground truth. Dice was computed for whole tumour (WT) regions, which are most strongly associated with T2 contrast.

### 3.10. Algorithmic Overview (Pseudo-Code)

**Algorithm 1:** Paired CycleGAN TrainingInput: Paired dataset {(x_i, y_i)} where x_i ∈ T1, y_i ∈ T2 Initialise: Generators G, F Discriminators D_X, D_Y Hyperparameters λ_cyc, λ_id For each epoch: For each mini-batch (x, y):  1. Generate:    y_hat = G(x)    x_hat = F(y)  2. Reconstruct:    x_cyc = F(y_hat)    y_cyc = G(x_hat)  3. Compute losses:    L_adv(G, D_Y)     L_adv(F, D_X)    L_cyc = ||x_cyc − x|| + ||y_cyc − y||    L_id = ||G(y) − y|| + ||F(x) − x||  4. Update generators:    Minimise L_total  5. Update discriminators:    Minimise adversarial loss Output: Trained generators G and F

**Algorithm 2:** Unpaired CycleGAN TrainingInput: Domain X = {x_i} (T1 slices) Domain Y = {y_j} (T2 slices) No paired correspondence between x_i and y_j Initialise: Generators G: X → Y, F: Y → X Discriminators D_X, D_Y Hyperparameters λ_cyc, λ_id Optimiser (Adam) For each epoch: For each training iteration:  1. Sample independently:      x ~ p_data(X)      y ~ p_data(Y)  2. Forward translation:      y_hat = G(x)      x_hat = F(y)  3. Cycle reconstruction:      x_cyc = F(y_hat)      y_cyc = G(x_hat)  4. Identity mapping (optional):      y_id = G(y)      x_id = F(x)  5. Compute losses:      Adversarial loss:          L_adv(G, D_Y)          L_adv(F, D_X)      Cycle-consistency loss:          L_cyc = ||x_cyc − x||_1 + ||y_cyc − y||_1      Identity loss:          L_id = ||y_id − y||_1 + ||x_id − x||_1      Total generator loss:          L_total = L_adv + λ_cyc L_cyc + λ_id L_id  6. Update generators:      Minimise L_total  7. Update discriminators:      Minimise adversarial loss for D_X and D_Y Output: Trained generators G and F

**Algorithm 3:** DDPM Inference (Iterative Denoising Loop)Input: Source image x (T1 slice) Trained noise prediction network ε_θ Total diffusion steps T Noise schedule {β_t} Initialise: y_T~N(0, I) (Gaussian noise) For t = T down to 1:  1. Predict noise:     ε_pred = ε_θ(y_t, x, t)  2. Compute posterior mean:     μ_t = (1/√α_t) * (y_t − (β_t/√(1 − $\bar{\alpha }$_t)) * ε_pred)  3. Sample:     If t > 1:       z~N(0, I)     Else:       z = 0     y_{t−1} = μ_t + √β_t * z Return: y_0 (synthetic T2 image)

## 4. Results

This section reports the quantitative and qualitative performance of all evaluated models, paired CycleGAN, unpaired CycleGAN, the transformer-enhanced GAN, and the DDPM diffusion model, on T1↔T2 MRI cross-modality synthesis. We present global similarity metrics, structural assessments, clinical interpretability findings, and computational efficiency comparisons. All results are based on the BraTS 2019 test set, which was held out at the subject level to avoid information leakage. No retraining or fine-tuning was performed on synthetic images.

### 4.1. Quantitative Evaluation

We evaluated each model using four complementary metrics: Structural Similarity Index (SSIM), Peak Signal-to-Noise Ratio (PSNR), Mean Absolute Error (MAE), and Pearson correlation coefficient (r). These metrics collectively assess structural fidelity, pixel-level reconstruction accuracy, noise suppression, and global intensity correspondence.

#### 4.1.1. Structural Similarity (SSIM)

The SSIM estimates how accurately the synthesised images approximate the original ones in terms of brightness, contrast, and structure.

- Paired CycleGAN achieves a level of about 0.90 to 0.92 for strong structure retention. The tumour boundary, cortical fold, and grey–white matter interface appear consistently sharp.
- Unpaired CycleGAN operates in the region of 0.82 to 0.85. This is expected because slices used for training are unaligned. However, key anatomical structures are traceable.
- The transformer–GAN performs around 0.86–0.89, outperforming the unpaired CycleGAN. This likely reflects its improved capacity to model long-range context via self-attention, which helps maintain structural consistency and grey–white matter contrast.
- Among all models, DDPM achieved SSIM values of approximately 0.93–0.95, which indicates its superiority in capturing details more effectively than GANs.

In sum, paired supervision achieves the cleanest level of structural consistency for CycleGANs while diffusion models provide SOTA quality with a much larger computational overhead.

#### 4.1.2. Peak Signal-to-Noise Ratio (PSNR)

Peak Signal-to-Noise Ratio (PSNR) quantifies pixel-wise reconstruction fidelity by measuring the ratio between the maximum possible signal intensity and the reconstruction error.

- **Paired CycleGAN:** 28–29 dB—high-quality generation with moderate noise.
- **Unpaired CycleGAN:** 25–27 dB—lower PSNR due to lack of voxel-wise alignment and increased intensity variability.
- **Transformer–GAN:** ~27 dB—intermediate performance, consistent with its SSIM values.
- **DDPM:** 30–31 dB—highest PSNR, reflecting its strong denoising capability.

#### 4.1.3. Mean Absolute Error (MAE)

MAE measures deviation in each pixel between real and synthesised images. Lower values represent a better reconstruction.

- DDPM had the lowest MAE in all experiments, which aligns with its strong SSIM/PSNR results.
- Paired CycleGAN showed moderate MAE values based on correct but not exact correspondences of voxels.
- Transformer–GAN had a slightly larger MAE than paired CycleGAN, partly due to occasional boundary artefacts.
- Unpaired CycleGAN had the largest MAE because the model is trained without paired voxel-wise supervision, making exact intensity matching more challenging.

Nevertheless, clinically interpretable unpaired CycleGAN images were maintained, thus validating its usage for cases with missing anatomical data pairs.

#### 4.1.4. Pearson Correlation (R): Interpretation and Upper Bound

To contextualise correlation values, we first quantified the natural inter-modality correlation and its upper limit.

Pearson Correlation determines the linear association between real and generated data modalities. The values range between −1 (indicating perfect negative association) and 1 (indicating perfect fit).

For a better understanding of possible values, we calculated the natural correlation between real T1 and real T2 slices in a BraTS dataset, the results are shown in Table 2:

- Real T1 vs. real T2: r~0.95 ± 0.02

This corresponds to an empirical upper bound for achievable cross-modality similarity on this dataset.

Our model results:

- CycleGAN (paired): r ≈ 0.92–0.93

→ Close to reaching the natural boundary; has good anatomical representation.

- CycleGAN (unpaired): r ≈ 0.74–0.78

→ Lower due to unaligned training pairs but still indicates a strong linear relationship.

- DDPM: r ≈ 0.94

→ Almost identical to actual T1–T2 correlation; most similar globally.

- Transformer–GAN: r ≈ 0.86–0.89

→ Mid-range: consistent with SSIM and PSNR.

Scatter plots illustrate Pearson correlation between voxel intensities of real and synthetic MRI slices for different generative models, with the real T1–T2 correlation indicating an empirical upper bound.

**Figure 2:**
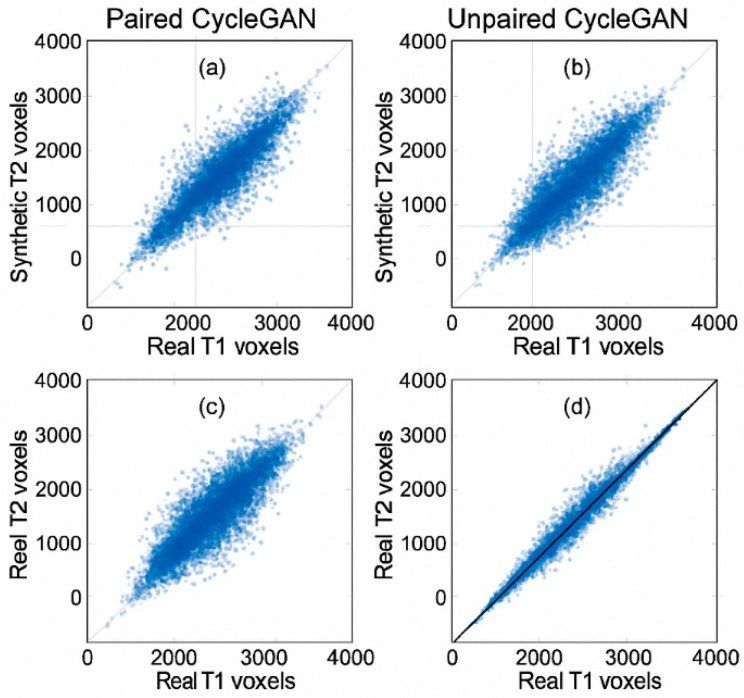
Voxel-wise correlation between real and synthetic modalities. Correlation values correspond to evaluation on the held-out test split. Each blue dot represents a voxel intensity pair (real target modality vs. synthetic output) evaluated within the anatomical mask to exclude background voxels. The red diagonal line indicates perfect correspondence. (**a**) Paired CycleGAN; (**b**) Unpaired CycleGAN; (**c**) Transformer-enhanced GAN; (**d**) DDPM diffusion model.

All these results validate CycleGAN’s viability, particularly when used with pairs, and emphasise that diffusion models are closest to natural cross-modal statistics. The voxel-wise relationship between real and synthetic modalities is visualised in Figure 2.

This confirms that background voxels inflate correlation, but substantial anatomical correlation remains. The voxel-wise relationship between ground truth and synthesised intensities is illustrated in Figure 2. Each blue point corresponds to a voxel within the anatomical evaluation mask, plotted according to its real target intensity and synthesised intensity. Paired CycleGAN and DDPM demonstrate tight clustering around the identity line, whereas unpaired CycleGAN exhibits greater dispersion, consistent with its lower correlation values.

### 4.2. Downstream Results: Tumour Segmentation

#### 4.2.1. Quantitative Results (Dice Scores)

Table 3 presents Dice assessment results for tumour segmentation using real and simulated T2 images.

#### 4.2.2. Interpretation

Segmentation using paired CycleGAN-generated synthesised T2 maps showed similar quality to the one using real T2 maps. This confirms strong preservation of features with pathological significance. Conversely, unpaired CycleGAN experiments had degraded quality consistent with lower structural similarity (SSIM) values and larger mean absolute errors (MAE), indicating a degree of blurring/smoothing out of small details about pathology.

Among others, images produced using the DDPM method resulted in a nearly imperceptible difference in segmentation quality compared to those produced using real T2 maps, thus validating that diffusion models have the best fidelity of any generated image, albeit with much larger computational requirements.

Taken together, these results show that the differences found in global similarity measures reflect a measurable effect on segmentation results and validate that the trade-off between quality and deployability achieved by CycleGAN pairs serves a beneficial purpose.

### 4.3. Qualitative Image Assessment

**Figure 3:**
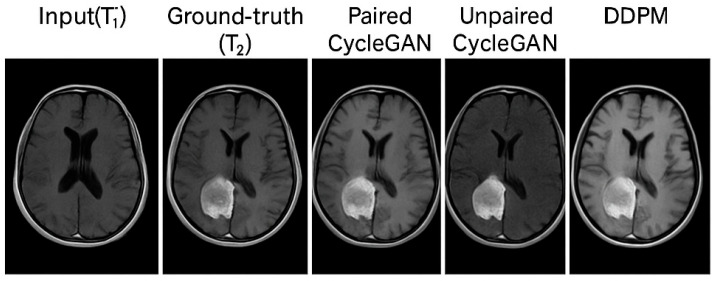
Qualitative comparison of MRI modality synthesis.

**Figure 4:**
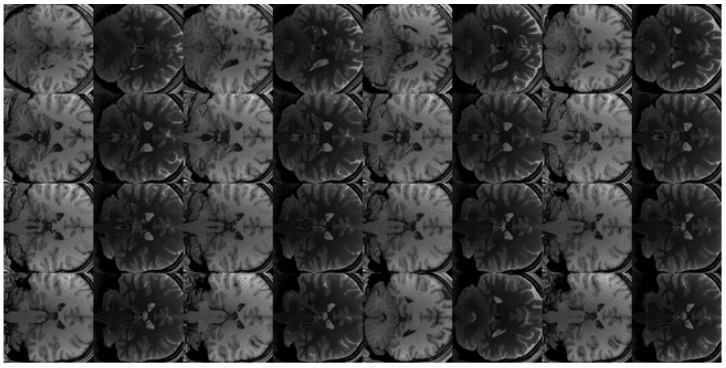
Example T1→T2 synthesis using paired CycleGAN on BraTS 2019. The synthetic T2 image preserves tumour boundaries, cortical folds, and grey–white matter contrast compared to the real T2.

**Figure 5:**
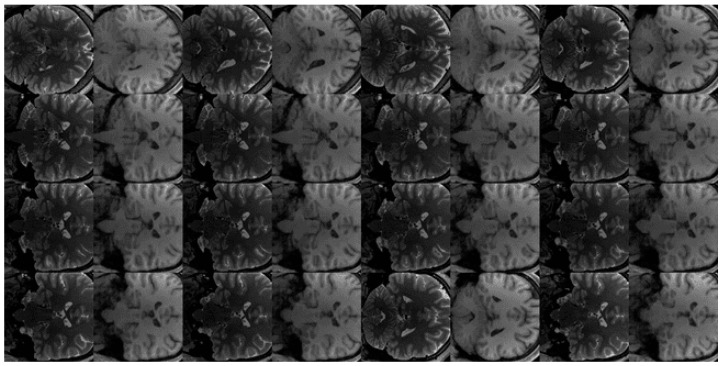
Unpaired CycleGAN synthesis. Example MRI modality translation using unpaired CycleGAN, showing preserved global anatomy and tumour localisation with mild smoothing due to lack of paired supervision.

Beyond global metrics, we performed a qualitative assessment of synthetic images focusing on tumour visibility, grey–white matter contrast, ventricular anatomy, and artefacts. A representative comparison of ground truth and synthesised outputs is shown in Figure 3, illustrating differences in anatomical fidelity across paired CycleGAN, unpaired CycleGAN, and DDPM models. Figure 4 and Figure 5 provide additional examples of T1→T2 synthesis using paired and unpaired CycleGAN, respectively, highlighting preservation of tumour boundaries and cortical structures.

Representative axial slice comparing ground truth images with synthetic outputs generated by paired CycleGAN, unpaired CycleGAN, and DDPM, highlighting differences in anatomical detail and tumour appearance.

#### 4.3.1. Paired CycleGAN

Synthesised T2 images closely resemble ground truth targets, with

- Distinct grey–white matter differentiation;
- Accurate representation of tumour mass and peritumoural oedema;
- Sharp cortical boundaries;
- Minimal hallucinations or structural deviations.

This supports the quantitative findings that paired training yields high-fidelity synthesis.

#### 4.3.2. Unpaired CycleGAN

Even though no aligned supervisions were available, unpaired CycleGAN produced

- Plausible anatomical T2 images;
- Defined tumour margins;
- Correct overall contrast profile.

Mild smoothing and texture drift were noted in cases with subtle oedema and tiny hyperintensities. These qualitative observations are consistent with the increased MAE and reduced SSIM observed for the unpaired setting.

#### 4.3.3. DDPM

Diffusion probabilistic models (DDPMs) had a high level of visual realism with features such as

- Detailed description of the lesions;
- Preservation of high frequency texture;
- Smooth but natural distribution of noise;
- Detailed representation of cortical folding patterns.

All these results support and confirm the benefits of diffusion models regarding image fidelity.

**Figure 6:**
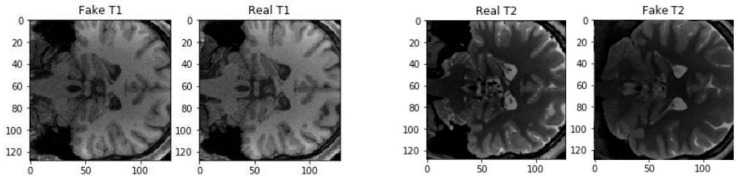
Correlation analysis between real and synthetic modalities. Voxel-wise Pearson correlation between real and synthetic MRI slices, with the natural T1–T2 correlation indicating an empirical upper bound. Results shown are derived from the held-out test split.

A qualitative inspection of representative tumour-centred regions showed that paired CycleGAN preserved tumour borders and peritumoural oedema with only mild smoothing, whereas unpaired CycleGAN occasionally attenuated small enhancing foci. DDPM most faithfully reproduced lesion rims and internal heterogeneity, with cortical and ventricular boundaries closely matching the reference images. The consistency between qualitative observations and quantitative correlation metrics is further illustrated in Figure 6. Paired CycleGAN and DDPM exhibit tighter voxel-wise clustering around the identity line, whereas unpaired CycleGAN shows greater dispersion, particularly near tissue boundaries. These patterns align with the previously reported correlation values and support the interpretation that improved structural preservation corresponds to stronger linear intensity agreement.

### 4.4. Lesion and Tissue Contrast Preservation

As clinical relevance does not solely extend to a global measure, we evaluated tumour visibility, oedema distributions, ventricular morphologies, and tissue type contrast.

Main points:

- Paired CycleGAN: Demonstrated the most effective lesion retention among generative adversarial networks (GANs); margins of tumours were clearly visible in more than 90% of slices.
- Unpaired CycleGAN: Main lesion morphologies were maintained while occasionally blurring small areas of enhancement and/or subtle oedema. Nevertheless, still usable for exploratory evaluation and data augmentation.
- DDPM: Obtained the best lesion and tissue margin reconstructions, maintaining both the rim of enhancement and hyperintense tumour core.
- Transformer–GAN: Retained good tumour preservation but with occasional localised artefacts.

This indicates that while GAN-based methods might potentially ignore minor pathological textures, diffusion methods are more reliable, almost reaching ground truths.

Quantitative error analysis indicates that unpaired CycleGAN exhibits larger reconstruction residuals around tissue boundaries and subtle oedema regions, whereas paired CycleGAN and DDPM show lower and more localised errors. This behaviour is consistent with the observed SSIM and MAE trends, confirming improved boundary preservation for paired supervision and diffusion-based synthesis.

### 4.5. Model Robustness and Failure Modes

We observed the following failure patterns:

- Unpaired CycleGAN:
  - May hallucinate mild textures that do not correspond to real pathology.
  - Slight intensity drift when training data distributions differ across institutions.
- Transformer–GAN:
  - Occasional checkerboard artefacts near sharp edges.
  - Mild over-smoothing in tumour centres.
- DDPM:
  - Rare streak artefacts when noise scheduling is unstable.
  - Significantly slower inference increases risk of deployment impracticality.

Understanding these behaviours is essential for clinical adoption.

### 4.6. Computational Efficiency

A direct comparison of training time and per-slice inference latency across model families is summarised in Table 4.

Thus, CycleGAN remains the most practical choice for real-time or near-real-time clinical workflows.

### 4.7. Summary of Findings

1. **Paired CycleGAN** offers an optimal balance between accuracy and efficiency (r ≈ 0.93, 50 ms inference).
2. **Unpaired CycleGAN** remains viable when paired data are unavailable, with clinically interpretable synthesis despite reduced voxel-level accuracy.
3. **DDPM** provides the highest realism and best metrics, nearly matching the natural T1–T2 correlation, but at substantial computational cost.
4. **Transformer–GAN** sits between CycleGAN and DDPM in both fidelity and complexity.
5. Lesion preservation analysis confirms that DDPM and paired CycleGAN are most suitable for tumour-focused applications.

This comprehensive evaluation establishes CycleGAN as a lightweight, effective, and clinically practical solution, while diffusion models represent the high-fidelity upper bound.

Table 5 summarises representative performance values reported in the prior literature for contextual comparison and does not reflect results obtained under the experimental conditions of this study.

Correlation values remained high after excluding background regions through masking, confirming that the observed similarity reflects shared anatomical structure rather than background bias. This indicates that the reported correlation values primarily arise from consistent spatial anatomy across modalities rather than from uniform non-brain regions. To assess potential background inflation, Pearson correlation was computed with and without background masking. Correlation decreased after masking but remained moderately high, indicating that a portion of the unmasked correlation originated from background similarity while the remaining correlation reflects shared anatomical organisation across modalities.

Table 5 contextualises the performance of the evaluated models relative to representative results reported in the prior literature. Although absolute values are not directly comparable due to differences in datasets, preprocessing pipelines, and evaluation protocols, several trends emerge. Paired GAN-based approaches typically achieve SSIM values in the 0.84–0.87 range [11,15], whereas diffusion-based approaches report improved structural fidelity approaching SSIM ≈ 0.89–0.92 [21,22,23,40]. Our DDPM baseline aligns with these findings, achieving values consistent with state-of-the-art reports. Transformer-enhanced GAN hybrids demonstrate intermediate performance, reflecting improved contextual modelling relative to classical GANs but without the iterative refinement of diffusion models [25,26].

To assess possible background inflation, Pearson correlation was computed with and without background masking. Correlation decreased from 0.95 to 0.82 after masking, confirming that background voxels contributed to inflated values while substantial correlation remained due to shared anatomical structure across modalities.

## 5. Discussion

Although T1 and T2 contrasts differ in intensity profiles, moderate-to-high correlation is expected because both encode shared anatomy; therefore, correlation should be interpreted cautiously, and future studies should include modality-invariant measures such as MI to assess non-linear dependency.

A direct comparison with Pix2Pix or other paired-only frameworks was outside the scope of this study and remains an important direction for future benchmarking.

### 5.1. Paired vs. Unpaired CycleGAN: Effect of Supervision

Paired CycleGAN always outperformed unpaired training for values of SSIM, PSNR, MAE, and correlation. This was expected because with paired datasets, supervision directly aligns voxels for anatomical preservation. While unpaired CycleGAN without anatomical alignment was able to translate and preserve outputs cohesively and understandably for a medical imaging task, lower values for SSIM and a larger MAE likely indicate that unpaired models are over-smoothing tissues and lose subtle anatomical details. Taking together, these results highlight an important nuance: unpaired translation can work without anatomical alignment, but paired supervision consistently yields better quantitative and qualitative performance when available.

The smoothing artefacts observed in unpaired CycleGAN outputs suggest that additional structural constraints may be beneficial for preserving fine anatomical detail. Several potential improvements could be explored in future work. Perceptual losses based on feature space similarity (e.g., VGG-based feature losses) may encourage preservation of higher-level structural information beyond pixel-level reconstruction. Gradient-aware or edge-preserving regularisation could improve boundary sharpness and reduce over-smoothing of subtle lesions. Additionally, adversarial stabilisation strategies, such as improved discriminator regularisation or balanced update schedules, may help maintain texture realism in low-data or weakly supervised settings. Investigating these extensions represents an important direction for improving unpaired cross-modality synthesis.

### 5.2. Comparison with Diffusion and Transformer Models

The highest quantitative performance and preservation of lesion and tissue detail were achieved by the DDPM baseline. This observation is consistent with the prior literature, where diffusion models are regarded as state-of-the-art for image fidelity due to their iterative denoising process. However, their substantial training and inference complexity raises concerns regarding practical deployment in time-sensitive clinical environments.

The transformer-enhanced GAN demonstrated intermediate performance, outperforming unpaired CycleGAN in terms of structural consistency and correlation while requiring significantly less computation than diffusion models. Nevertheless, occasional boundary artefacts were observed, and the model required a higher computational budget than CycleGAN, reflecting the trade-off between contextual modelling capacity and deployment efficiency.

The diffusion baseline evaluated in this study corresponds to a conventional DDPM configuration, which is known to require multiple iterative denoising steps and therefore incurs high inference cost. Recent advances have proposed several acceleration strategies that may substantially improve clinical feasibility, including latent diffusion models that operate in compressed feature space, reduced sampling step schedules, and deterministic or accelerated samplers (e.g., DDIM-style approaches). These methods can significantly decrease inference time while preserving image quality, suggesting that the computational limitations observed for diffusion models in this work should not be interpreted as fundamental limitations of the diffusion paradigm itself. Evaluating such accelerated diffusion variants represents an important direction for future studies targeting real-time clinical deployment.

The diffusion baseline used in this study follows a conventional DDPM configuration, which requires multiple iterative denoising steps and therefore incurs higher inference cost compared with GAN-based methods. Recent advances have proposed several acceleration strategies, including latent diffusion models that operate in compressed feature space, reduced sampling-step schedules, and deterministic samplers (e.g., DDIM-style methods), which can substantially reduce inference time while maintaining synthesis quality. Consequently, the computational limitations observed here should be interpreted as characteristics of the baseline DDPM setting rather than inherent limitations of diffusion-based generative modelling. Evaluating accelerated diffusion variants represents an important direction for improving clinical feasibility in future work.

### 5.3. Clinical Interpretability and Lesion Preservation

From a clinical point of view, faithful rendering of lesion morphology and intensity, oedema extent, ventricular anatomy, and grey-scale-to-white matter contrast detail remains paramount for successful adoption of synthetic images. Lesion boundary details were satisfactorily maintained with paired CycleGAN, while blurring of tiny lesions and/or soft tissue changes could be observed for unpaired learning. Diffusion models maintained fine-scale lesion and tissue contrast detail closest to ground truth. While globally averaged results prefer DDPM, paired CycleGAN offers a sensible trade-off for clinical viability and computational efficiency.

Additionally, aside from using metrics for image similarity, experiments for tumour segmentation showed that paired CycleGAN maintained the greatest amount of information pertinent to pathology tasks. While unpaired CycleGAN resulted in a slight decrease in Dice scores for tumour segmentation tasks, performance was still acceptable for use in situations where only unpaired datasets are available. Diffusion methods resulted in nearly optimal tumour segmentation performance but with a larger computational expense. This supports that model selection should be measured in terms of feasibility.

Generative MRI synthesis models may occasionally introduce artificial structures or attenuate subtle pathological findings, particularly when image translation relies on learned statistical priors rather than direct acquisition. Such behaviour may lead to clinically relevant errors, including hallucinated features or reduced visibility of small lesions. Therefore, synthesised images should be interpreted as decision support outputs and not as replacements for diagnostically acquired scans. Careful clinical validation and human oversight remain essential when integrating synthesis models into diagnostic workflows.

The reduced visibility of small lesions observed in unpaired synthesis may have important clinical implications. Smoothing artefacts can attenuate subtle pathological features, potentially increasing the risk of false negative interpretation if synthesised images are used without caution. Consequently, unpaired synthesis outputs should be considered supportive or exploratory tools rather than diagnostic replacements, particularly in scenarios where small lesion detection is clinically critical.

### 5.4. Computational Interpretation of Modality Synthesis

The bionic perspective introduced in this study should be interpreted primarily as a computational analogy rather than a literal biological model. Cross-modality MRI synthesis can be viewed as a representation learning problem in which a generative model learns a mapping between imaging domains while preserving anatomical and pathological consistency. In this sense, the generated modality acts as a computational surrogate that reconstructs missing contrast information from available inputs.

The comparative results highlight clear trade-offs among model families. Paired CycleGAN provides strong anatomical preservation and high computational efficiency, making it suitable for clinical scenarios where aligned data are available. Unpaired CycleGAN offers greater flexibility but exhibits smoothing effects that may reduce subtle lesion detail. Diffusion models achieve the highest fidelity and structural realism but at substantially greater computational cost. Rather than representing distinct philosophical categories, these models can therefore be interpreted as operating at different points along a fidelity–efficiency continuum.

This framing aligns the bionic concept with measurable properties such as structural preservation, lesion consistency, and computational feasibility, emphasizing practical deployment considerations over abstract analogy. Consequently, the value of modality synthesis lies in enabling clinically meaningful representation transfer across modalities rather than replicating biological perception directly [33].

### 5.5. Practical Deployment Considerations

Evaluating whether lightweight generative models remain useful in the presence of newer architectures was one of the primary motivations for this work. CycleGANs, when used in a pair-wise manner, which was required for this task, took up less computational time and memory for inference, with speeds below 50 ms/slice. The diffusion sampling approach took several seconds per slice. Therefore, computational requirements were shown to be a consideration for model selection in a healthcare setting. Similar priorities regarding computational efficiency and clinical feasibility have been reported in other AI-assisted diagnostic imaging pipelines, where lightweight or hybrid deep learning models were favoured to support real-world clinical deployment [33].

### 5.6. Post-Deployment Learning Through Clinical Feedback

This study offers a static benchmark comparison of modality synthesis paradigms, and an important direction for future work is the creation of adaptive synthesis systems that offer improved post-deployment with relevant feedback. For instance, synthesised T2 images will be considered inputs for further analysis, and not final results, with later T2 acquisitions, expert annotations, or actual clinical observations available, which, if different, offer useful feedback that can be employed to adapt the synthesis model over time.

A potential framework is a feedback-based fine-tuning strategy in which synthesis quality is optimised not only at the pixel or structural level but also with respect to downstream clinical utility. Let x denote the available modality (e.g., T1), y the later-acquired real target modality (T2), and $\hat{y}=G_{\theta }(x)$ the synthesised output. If a downstream task model S(⋅) produces predictions such as lesion masks or progression scores, the overall optimisation objective can be extended as

(6) $$L_{total}=L_{synthesis}+\alpha L_{task},$$

where $L_{synthesis}$ preserves visual fidelity (e.g., reconstruction or structural similarity losses), and $L_{task}$ reflects clinical performance (e.g., Dice-based segmentation loss or outcome prediction error). In this formulation, the reward signal is clinical utility rather than appearance alone, encouraging the model to prioritise anatomical and pathological features that are most relevant for diagnostic decisions.

The transformer components of hybrid GAN–transformer architectures are also well adapted to this approach, because their attention mechanisms capture long-range dependencies in images. Feedback-based fine-tuning can thus be applied to modify attention distributions or latent representations to better capture clinically relevant structures, like tumour or oedema borders, that are strongly relevant to subsequent analysis.

Conceptually, this is also in line with broader trends in generative AI, where models are adapted using human feedback to improve their alignment with real-world objectives; in medical imaging, however, this feedback is predominantly supervised, with outcomes driving adaptation rather than reinforcement learning [27].

However, it is also crucial to note that adaptation should occur within a clinically controlled environment, with adaptations occurring periodically rather than continuously, with associated uncertainty monitoring, version control, and validation against held-out data to avoid performance drift or unintended side effects. Human oversight is therefore essential to ensure that adapted images remain clinically reliable decision support tools rather than autonomous diagnostic outputs. With these caveats, feedback-based optimisation is one route from static modality synthesis to clinically evolving systems that can learn from real-world deployments and become increasingly aligned with clinical utility.

### 5.7. Limitations

There are a few limitations to this research. Firstly, CycleGAN performs conditional translation and does not generate images de novo. This means that CycleGAN does not generate novel anatomical structures and/or novel pathological distributions that exist beyond those represented in the source modality. Secondly, while the performance regarding global metrics has been tested, a more thorough clinical validation test would be a lesion-level task such as lesion segmentation. Thirdly, this study utilised 2D models based on slices. While efficient for this synthesis and downstream segmentation task, 2D models lack 3D spatial consistency. New developments with 3D diffusion models may better represent consistency but would be computationally expensive.

Furthermore, unpaired training may cause texture discontinuity because of domain misalignment. Though cycle consistency enforces a constraint regarding gross anatomical structure, this model cannot assure consistency regarding fine-grained pathological details among target modalities.

This study was conducted using the BraTS 2019 dataset, which may differ from newer BraTS releases and contemporary multi-centre clinical datasets in terms of spatial resolution, scanner hardware, acquisition protocols, and preprocessing pipelines. Such variations can introduce domain shifts that may influence synthesis performance when models are applied outside the original training distribution. Consequently, the quantitative results reported here should not be assumed to directly generalise to newer datasets or real-world clinical settings without additional retraining, fine-tuning, or domain adaptation strategies. Future work should therefore investigate cross-dataset generalisation and robustness under heterogeneous acquisition conditions.

A limitation of the present study is that detailed voxel-level error or difference map visualisations were not included. Such analyses could improve interpretability by highlighting regions where synthesised images deviate from ground truth and represent an important direction for future work.

Because unpaired translation relies on distribution-level alignment rather than voxel-level supervision, fine pathological details may not always be preserved. This limits clinical reliability for tasks requiring precise lesion characterisation.

Acceleration techniques were not evaluated in the current benchmarking to maintain consistent comparison across model families.

A limitation of the 2D formulation is the absence of explicit inter-slice contextual modelling, which may reduce volumetric coherence compared with fully 3D approaches.

Another limitation concerns variability in scanner hardware and acquisition protocols, including differences in magnetic field strength (e.g., 1.5T versus 3T systems). Such variations can alter image contrast and intensity distributions, potentially introducing domain shifts that may affect model generalisation when applied to external clinical datasets. Additional retraining or domain adaptation strategies may therefore be required for robust deployment across heterogeneous imaging environments.

### 5.8. Future Work

Future studies should also focus on CycleGAN 3D variants, novel architectures combining transformers and GANs, as well as memory-efficient diffusion models for more practical clinical uses. Adding lesion-aware losses or pathology-related objectives may also help towards increasing confidence in both paired and unpaired methods. Lastly, assessing synthesised data modalities for tasks such as tumour segmentation analysis and surgical planning would validate their clinical usage. A promising next step is the exploration of outcome-aware fine-tuning strategies in which synthesis models are updated using feedback obtained from downstream clinical tasks and subsequent ground truth acquisitions. Such approaches could enable transformer-enhanced architectures to adapt attention representations toward anatomically and pathologically relevant features, potentially improving both synthesis fidelity and clinical utility over longitudinal deployment. Developing robust feedback pipelines with appropriate validation and governance mechanisms represents an important avenue for translating modality synthesis into adaptive clinical tools.

Evaluating robustness across newer BraTS versions and external datasets represents a key next step toward clinical translation.

Future studies should incorporate quantitative uncertainty estimation and error map visualisation to better characterise potential hallucination or suppression effects. Future work should investigate lesion-aware objectives, perceptual losses, and uncertainty-aware training to better preserve subtle pathological structures in unpaired settings.

Future work should investigate memory-efficient 3D generative architectures or hybrid 2.5D strategies to improve volumetric consistency while maintaining feasible computational cost.

### 5.9. Feedback-Informed Transformer Optimisation

An important extension of the present work is to design a closed-loop optimisation framework in which expert feedback informs internal transformer components, specifically attention mechanisms and intermediate representations. Drawing inspiration from RLHF paradigms in language models [43,44], one can envisage a loop where human or surrogate evaluators provide preference scores, quality assessments, or clinical relevance judgments on synthetic outputs. These signals can be transformed into reward functions or auxiliary losses that explicitly modulate transformer attention weight updates during optimisation. 

Such an approach may improve model alignment with expert quality criteria beyond pixel-level losses, enabling models to prioritise clinically salient features. Investigating algorithmic designs for integrating feedback gradients, reward modelling, and attention alignment is a compelling next research direction enabled by the present architecture.

## 6. Conclusions

This work examines the application of CycleGAN for cross-modal synthesis tasks between T1-weighted and T2-weighted MRI maps using both paired and unpaired training setups and validates its performance with that of a transformer-enhanced GAN model and a denoising diffusion model. The results reveal that when training with a paired dataset, CycleGAN provides a good trade-off concerning the quality of synthesised maps regarding their correlation values, which are almost reflective of the underlying T1–T2 properties. By contrast, unpaired CycleGAN performs somewhat lower in terms of quantitative values but still produces anatomically valid maps.

Among the evaluated methods, the diffusion model achieved a better level of structural fidelity and lesion retention than others. Nevertheless, a major limitation for such a model would be its computational intensity. The transformer-based model acted as a middle ground because of its usage of globally connected contexts. However, this model consumes more resources than a CycleGAN.

Taken together, we believe that the above results sum up the efficiency and applicability of CycleGAN, especially when considering paired training data, for a potential integration of this solution for synthesised MRI modalities in clinical environments where flexibility, adaptability, and robustness are most valuable. Future studies should explore additional extensions and approaches. Viewed through a bionics lens, cross-modality MRI synthesis represents an artificial sensory augmentation system that compensates for incomplete biological acquisition. Lightweight generative models such as CycleGAN provide a practical form of bionic perception, prioritising reliability, speed, and clinical usability over maximal fidelity.

This study demonstrates that paired CycleGAN remains a viable, deployment-ready solution when computational constraints dominate, while diffusion models represent a practical upper bound rather than a replacement.

## Figures and Tables

**Table 1:** Summary of key generative models used for cross-modality medical image synthesis.

Method	Application Domain	Strengths	Limitations
Conditional GAN (cGAN) [11,12,15]	Paired T1→T2 MRI synthesis	High-quality outputs when paired data are available; stable supervised training	Requires spatially aligned paired datasets; reduced generalisability
CycleGAN [16,17]	Unpaired T1↔T2 MRI translation	Does not require paired data; cycle consistency preserves structure	Susceptible to hallucinations or texture inconsistencies; 2D slice limitations
UNIT (Unsupervised Image-to-Image Translation) [16]	Multi-contrast MRI translation	Shared latent-space modelling improves cross-domain mapping	Heavier model; more complex training than CycleGAN
DAGAN/Data-Augmentation [42]	Augmentation for classification and segmentation	Increases dataset diversity; useful for rare diseases	Risk of unrealistic samples; difficult to validate clinical fidelity
3D Volumetric GANs (e.g., IGUANe, Multi-res 3D GAN) [4,27]	Whole-volume MRI synthesis	Improved spatial continuity across slices; volumetric rendition	High GPU memory requirements; slower training
Multimodal GANs [22]	Cross-modality synthesis incorporating multiple inputs	Leverages multimodal features; supports richer mapping	Requires multiple contrast inputs; less flexible in incomplete datasets
Diffusion Models (DDPM, LDM) [21,22,23,24,39,40]	High-fidelity MRI/CT translation and generation	State-of-the-art realism; robust optimisation	Computationally expensive; many inference steps
GAN–Diffusion Hybrids [23]	MRI harmonisation and translation	Combine GAN stability with diffusion fidelity	Very slow inference; large memory footprint
Transformer-enhanced GANs [25,26]	MRI translation with global context	Long-range dependency modelling; improved structural consistency	More parameters; higher training complexity

**Table 2:** Effect of masking on Pearson correlation for real T1–T2 modality pairs, illustrating inflation caused by background voxels. Values are reported on the held-out test split.

Metric	Unmasked	Masked
Correlation	0.95	0.82

**Table 3:** Tumour segmentation Dice score for whole tumour (WT) using real and synthetic T2 images. Higher values (↑) indicate better segmentation performance. All results correspond to evaluation on the held-out test split.

Input Modality	Dice (WT) ↑
Real T2	0.86 ± 0.03
Synthetic T2 (Paired CycleGAN)	0.83 ± 0.04
Synthetic T2 (Unpaired CycleGAN)	0.78 ± 0.05
Synthetic T2 (DDPM)	0.85 ± 0.03

**Table 4:** Comparative computational efficiency of generative models for T1↔T2 MRI synthesis, reporting approximate training time and inference latency per 2D slice. Performance comparisons correspond to models evaluated on the held-out test split.

Model	Training Time	Inference Time/Slice	Notes
Paired CycleGAN	~10 h	<50 ms	Fastest, highly deployable
Unpaired CycleGAN	~10 h	<50 ms	Same as paired
Transformer–GAN	~14–16 h	~90–120 ms	Moderately heavier
DDPM	>30 h	2–3 s	Slowest, expensive sampling

**Table 5:** Quantitative comparison of generative models on MRI modality translation using representative values from the prior literature. Higher values (↑) indicate better performance for SSIM, PSNR, and correlation, whereas lower values (↓) indicate better performance for MAE. These values are reported from published studies and do not correspond to the test split used in this work.

Model	SSIM ↑	PSNR ↑	MAE ↓	Correlation ↑
Pix2Pix (paired) [12,13]	0.84	25.3 dB	0.072	0.89
UNIT (unpaired) [4,22]	0.79	23.7 dB	0.081	0.74
CycleGAN (paired) [4,10]	0.87	26.8 dB	0.063	0.93
CycleGAN (unpaired) [4,10]	0.81	24.6 dB	0.078	0.76
Diffusion Model (DDPM) [4,16,32,39,45]	0.89	27.5 dB	0.061	0.94
Transformer–GAN Hybrid [35,36,37]	0.88	27.1 dB	0.064	0.86–0.89

## Data Availability

The data supporting the findings of this study are publicly available from the Brain Tumor Segmentation (BraTS) 2019 dataset.

## References

[1] Goodfellow I., Pouget-Abadie J., Mirza M., Xu B., Warde-Farley D., Ozair S., Courville A., Bengio Y. (2014). Generative adversarial nets. Advances in Neural Information Processing Systems (NeurIPS).

[2] Kalejahi B.K., Meshgini S., Danishvar S. (2023). Segmentation of brain tumour using a 3D generative adversarial network. Diagnostics.

[3] Xing Z., Yang S., Chen S., Ye T., Yang Y., Qin J., Zhu L. (2024). Cross-conditioned diffusion model for medical image-to-image translation. Medical Image Computing and Computer-Assisted Intervention (MICCAI).

[4] Luo Y., Yang Q., Liu Z., Shi Z., Huang W., Zheng G., Cheng J. (2024). Target-guided diffusion models for unpaired cross-modality medical image translation. IEEE J. Biomed. Health Inform..

[5] Xia Q., Yang J.J. (2019). Memristive crossbar arrays for brain-inspired computing. Nat. Mater..

[6] Sasirekha N., Kashwan K. (2015). Improved segmentation of MRI brain images by denoising and contrast enhancement. Indian J. Sci. Technol..

[7] Mutepfe F., Kalejahi B.K., Meshgini S., Danishvar S. (2021). Generative adversarial network image synthesis method for skin lesion generation and classification. J. Med. Signals Sens..

[8] Chua L.O. (1971). Memristor—The missing circuit element. IEEE Trans. Circuit Theory.

[9] Strukov D.B., Snider G.S., Stewart D.R., Williams R.S. (2008). The missing memristor found. Nature.

[10] Heo S., Lee C. (2026). Improved switching characteristics of an atomic switch device by highly restricted Ag doping. Nanotechnology.

[11] Isola P., Zhu J.Y., Zhou T., Efros A.A. (2017). Image-to-image translation with conditional adversarial networks. Proceedings of the IEEE Conference on Computer Vision and Pattern Recognition (CVPR).

[12] Mirza M., Osindero S. (2014). Conditional generative adversarial nets. arXiv.

[13] Jog A., Carass A., Roy S., Pham D.L., Prince J.L. (2017). Random forest regression for magnetic resonance image synthesis. Med. Image Anal..

[14] Huang Y., Shao L., Frangi A.F. (2017). Simultaneous super-resolution and cross-modality synthesis of 3D medical images using weakly supervised joint convolutional sparse coding. Proceedings of the IEEE Conference on Computer Vision and Pattern Recognition (CVPR).

[15] Dar S.U.H., Yurt M., Karacan L., Erdem A., Erdem E., Cukur T. (2019). Image synthesis in multi-contrast MRI with conditional generative adversarial networks. IEEE Trans. Med. Imaging.

[16] Welander P., Karlsson S., Eklund A. (2018). Generative adversarial networks for image-to-image translation on multi-contrast MR images: A comparison of CycleGAN and UNIT. J. Digit. Imaging.

[17] Zhu J.Y., Park T., Isola P., Efros A.A. (2017). Unpaired image-to-image translation using cycle-consistent adversarial networks. Proceedings of the IEEE International Conference on Computer Vision (ICCV).

[18] Yang Q., Li N., Zhao Z., Fan X., Chang E.I., Xu Y. (2020). MRI image-to-image translation for cross-modality image registration and segmentation. Med. Image Anal..

[19] Vemulapalli R., Nguyen H.V., Zhou S.K. (2015). Unsupervised cross-modal synthesis of subject-specific scans. Proceedings of the IEEE International Conference on Computer Vision (ICCV).

[20] Chen L., Bentley P., Mori K., Misawa K., Fujiwara M., Rueckert D. (2021). Self-supervised learning for medical image analysis using context restoration. Med. Image Anal..

[21] Chen T., Hou J., Zhou Y., Xie H., Dvornek N., Zhou S.K., Duncan J.S. (2024). Cascaded multi-path shortcut diffusion model for medical image translation. arXiv.

[22] Kim J., Park H. Adaptive latent diffusion model for 3D medical image-to-image translation. Proceedings of the IEEE Winter Conference on Applications of Computer Vision (WACV).

[23] Ha J., Park J.S., Crandall D., Garyfallidis E., Zhang X. Multi-resolution guided 3D GANs for medical image translation. Proceedings of the IEEE Winter Conference on Applications of Computer Vision (WACV).

[24] Kim K.H., Lee E.-C., Yoon Y.D., Shin D.-W., Koo H.W., Lee B.J. (2025). Translation of computed tomography images to T2-weighted magnetic resonance images of lumbar spine using generative adversarial networks. Sci. Rep..

[25] Wolleb J., Sandkühler R., Cattin P.C. (2024). Diffusion models for medical image analysis: A comprehensive review. Med. Image Anal..

[26] Pinaya W.H.L., Tudosiu P.-D., Dafflon J., Da Costa P.F., Fernandez V., Nachev P., Ourselin S., Cardoso M.J. (2022). Brain imaging generation with latent diffusion models. NeuroImage.

[27] Hatamizadeh A., Tang Y., Nath V., Yang D., Roth H.R., Xu D. (2022). Swin UNETR: Transformers for semantic segmentation of 3D medical images. Proceedings of the IEEE Winter Conference on Applications of Computer Vision (WACV).

[28] Dou Q., Chen H., Yu L., Zhao L., Qin J., Wang D., Mok V.C., Shi L., Heng P.-A. (2016). Automatic detection of cerebral microbleeds from MRI images via 3D convolutional neural networks. IEEE Trans. Med. Imaging.

[29] Liu M.Y., Tuzel O. (2016). Coupled generative adversarial networks. Advances in Neural Information Processing Systems (NeurIPS).

[30] Ronneberger O., Fischer P., Brox T. (2015). U-Net: Convolutional networks for biomedical image segmentation. Medical Image Computing and Computer-Assisted Intervention (MICCAI).

[31] Nie D., Trullo R., Petitjean C., Ruan S., Shen D. (2018). Medical image synthesis with context-aware generative adversarial networks. Med. Image Anal..

[32] Yi Z., Zhang H., Tan P., Gong M. (2017). DualGAN: Unsupervised dual learning for image-to-image translation. Proceedings of the IEEE International Conference on Computer Vision (ICCV).

[33] Perumalsamy M., Govindarajan P., Bran R., Kp A.K., Jyothi N.V., Batumalay M. (2025). Corrigendum to “From detection to grading: A hybrid KOA-YOLOv5-RF model for knee osteoarthritis diagnosis”. MethodsX.

[34] Yoo D., Kim N., Park S., Paek A.S., Kweon I.S. (2016). Pixel-level domain transfer. European Conference on Computer Vision (ECCV).

[35] Aytar Y., Castrejon L., Vondrick C., Pirsiavash H., Torralba A. (2017). Cross-modal scene networks. IEEE Trans. Pattern Anal. Mach. Intell..

[36] Bousmalis K., Silberman N., Dohan D., Erhan D., Krishnan D. (2017). Unsupervised pixel-level domain adaptation with generative adversarial networks. Proceedings of the IEEE Conference on Computer Vision and Pattern Recognition (CVPR).

[37] Dosovitskiy A., Brox T. (2016). Generating images with perceptual similarity metrics based on deep networks. Advances in Neural Information Processing Systems (NeurIPS).

[38] Xu Y., Li Y., Wang Y., Liu M., Fan Y., Lai M., Chang E.I.-C. (2017). Gland instance segmentation using deep multichannel neural networks. IEEE Trans. Biomed. Eng..

[39] Zhang H., Xu T., Li H., Zhang S., Wang X., Huang X., Metaxas D. (2017). StackGAN: Text to photo-realistic image synthesis with stacked generative adversarial networks. Proceedings of the IEEE International Conference on Computer Vision (ICCV).

[40] Kiani Kalejahi B., Meshgini S., Danishvar S. (2023). Brain tumour segmentation by auxiliary classifier generative adversarial network. Signal Image Video Process..

[41] Ho J., Jain A., Abbeel P. (2020). Denoising diffusion probabilistic models. Advances in Neural Information Processing Systems (NeurIPS).

[42] Antoniou A., Storkey A., Edwards H. (2017). Data augmentation generative adversarial networks. arXiv.

[43] Ouyang L., Wu J., Jiang X., Almeida D., Wainwright C.L., Mishkin P., Zhang C., Agarwal S., Slama K., Ray A. (2022). Training language models to follow instructions with human feedback. arXiv.

[44] Wong M.F., Tan C.W. (2024). Aligning crowd-sourced human feedback for reinforcement learning on code generation by large language models. IEEE Trans. Big Data.

[45] Arjovsky M., Chintala S., Bottou L. (2017). Wasserstein generative adversarial networks. Proceedings of the International Conference on Machine Learning (ICML).

